# Treating acute exacerbations of COPD with Chinese herbal medicine to aid antibiotic use reduction (Excalibur): a randomised double-blind, placebo-controlled feasibility trial

**DOI:** 10.3389/fphar.2023.1221905

**Published:** 2023-09-25

**Authors:** Merlin L. Willcox, Xiao-Yang Hu, Tom Oliver, Kerensa Thorne, Cherish Boxall, George He, Catherine Simpson, Becci Brotherwood, Alice O’Neil, Robert Waugh, Emma Tilt, Jeanne Trill, Neville Goward, Nick Francis, Michael Thomas, Paul Little, Tom Wilkinson, Jian-Ping Liu, Gareth Griffiths, Michael Moore

**Affiliations:** ^1^ Primary Care Research Centre, School of Primary Care, Population Sciences and Medical Education, Faculty of Medicine, University of Southampton, Southampton, United Kingdom; ^2^ Southampton Clinical Trials Unit, University of Southampton, Southampton, United Kingdom; ^3^ Phoenix Medical Ltd, Chelmsford, United Kingdom; ^4^ Patient and Public Representative, Southampton, United Kingdom; ^5^ School of Clinical and Experimental Sciences, Faculty of Medicine, University of Southampton, Southampton, United Kingdom; ^6^ Centre for Evidence-based Chinese Medicine, Beijing University of Chinese Medicine, Beijing, China

**Keywords:** COPD, Chinese herbal medicine, feasibility clinical trial, Shufeng Jiedu capsule, acute exacerbation of chronic obstructive pulmonary disease

## Abstract

**Background:** Although many acute exacerbations of COPD (AECOPD) are triggered by non-bacterial causes, they are often treated with antibiotics. Preliminary research suggests that the Chinese herbal medicine “Shufeng Jiedu” (SFJD), may improve recovery and therefore reduce antibiotic use in patients with AECOPD.

**Aims:** To assess the feasibility of conducting a randomised placebo-controlled clinical trial of SFJD for AECOPD in UK primary care.

**Methods:** GPs opportunistically recruited patients experiencing an AECOPD. Participants were randomised 1:1 to usual care plus SFJD or placebo for 14 days. Participants, GPs and research nurses were blinded to treatment allocation. GPs could prescribe immediate, delayed or no antibiotics, with delayed prescribing encouraged where appropriate. Participants were asked to complete a participant diary, including EXACT-PRO and CAT™ questionnaires for up to 4 weeks. Outcomes included recruitment rate and other measures of study feasibility described using only descriptive statistics and with no formal comparisons between groups. We also conducted qualitative interviews with recruited and non-recruited COPD patients and clinicians, analysed using framework analysis.

**Results:** Over 6 months, 19 participants (6 SFJD, 13 placebo) were recruited. Sixteen (84%) participants returned diaries or provided a diary by recall. Overall, 1.3 participants were recruited per 1,000 patients on the COPD register per month open. Median duration of treatment was 9.8 days in the intervention group vs 13.3 days in the placebo group. The main reason for discontinuation in both groups was perceived side-effects. in both groups. Point estimates for both the EXACT-PRO and CAT™ outcomes suggested possible small benefits of SFJD. Most patients and clinicians were happy to try SFJD as an alternative to antibiotics for AECOPD. Recruitment was lower than expected because of the short recruitment period, the lower incidence of AECOPD during the COVID-19 pandemic, patients starting antibiotics from “rescue packs” before seeing their GP, and workforce challenges in primary care.

**Conclusion:** Recruitment was impaired by the COVID-19 pandemic. Nevertheless, we were able to demonstrate the feasibility of recruiting and randomising participants and identified approaches to address recruitment challenges such as including the trial medication in COPD patients’ “rescue packs” and delegating recruitment to a central trials team.

**Clinical Trial Registration:** Identifier, ISRCTN26614726

## Introduction

The global burden of Chronic obstructive pulmonary disease (COPD) has increased steadily from 1990 to 2019, so that it is now the third leading global cause of death (WHO, 2020). In the UK, 1.8 million people have been diagnosed with COPD, and the COPD mortality rate is the third highest in Europe (Snell et al., 2016). Patients with COPD typically experience 1–2 “Acute exacerbations” (AECOPD) per year (Hurst et al., 2010), which are typically caused by acute viral infections on the background of chronic bacterial colonisation (Wilkinson et al., 2017). AECOPD are a major reason for healthcare consultations in primary care, hospital admissions, deterioration in function, and mortality in patients with COPD. In the UK, COPD is estimated to result in around 1.4 million GP consultations and 130,000 emergency hospital admissions each year, with an annual direct cost to the NHS of between £810 - £930 million. Over 70% of patients presenting with AECOPD in UK primary care are currently prescribed antibiotics (Butler et al., 2019), although only 44% of patients providing a baseline sputum sample had a bacterial pathogen isolated (unpublished data). Antimicrobial treatment in patients with COPD can reduce the infecting load without entirely eradicating organisms in the airways, leading to an increased risk of resistant bacteria (Miravitlles and Anzueto, 2013). Furthermore, over one-third of patients admitted to hospital with AECOPD are re-admitted within 90 days, and better interventions are needed to prevent these re-admissions (Kong and Wilkinson, 2020).

In China, many patients already use Traditional Chinese Medicine (TCM) for AECOPD, although there is limited evidence on the effectiveness of any of the TCM products for AECOPD (Chen et al., 2014). Although TCM has existed in China for millennia, it is only recently that standardised formulations have been patented. Shufeng Jiedu (SFJD) is one such standardised preparation which is licensed in China for the treatment of respiratory infections. It is available from 20,000 hospitals covering 298 cities all over China. In 2017–8, 23 million boxes were sold in China (Trill et al., 2022). A systematic review suggests that SFJD plus usual care (antibiotics and symptomatic treatments) is associated with a significant reduction in treatment failure, from 20.1% to 8.3% (11 trials; 815 patients; relative risk 0.43, 95% confidence interval [CI] 0.30 to 0.62; low certainty). Treatment failure was defined as no resolution or deterioration of symptoms after trial medication of any duration, or death (when explicitly stated, due to exacerbation) or additional course of antibiotics or another medication for the treatment of AECOPD (Xia et al., 2020). SFJD also reduced duration of hospital admission (2 trials; 79 patients; mean difference −4.35 days, 95% CI -5.28 to −3.43 days, low certainty) when compared with usual care. However, all included trials were at high risk of bias, due to lack of blinding and other factors (Xia et al., 2020). There was no evidence of a significant difference in adverse events between the intervention and control groups and there are no serious safety concerns (Trill et al., 2022). SFJD also seems to reduce duration of symptoms in COVID-19 (Sheng et al., 2022), upper respiratory tract infections (Zhang et al., 2021) and in community-acquired pneumonia, when given in addition to antibiotics (Zhang et al., 2022).

SFJD is a standardised combination of eight herbs (Table 1), all of which have been used in TCM for centuries, and all of which are available in the UK through consultation with a herbal practitioner (not restricted by the Medicines and Healthcare products Regulatory Agency (MHRA)). Each of these herbs has some evidence of safety and effectiveness for the treatment of respiratory infections (Yan et al., 2021; Wang et al., 2022). Compared to antibiotics, SFJD has many more mechanisms of action. It is not only antibacterial (Bao et al., 2016) but also antiviral, anti-inflammatory (Li et al., 2017) and immune modulating (Tao et al., 2014). Thus it has the potential simultaneously to address several of the causes of AECOPD, and so to reduce the emergence of resistant bacteria and the frequency of relapse after treatment with antibiotics.

**TABLE 1:** Composition of Shufeng Jiedu.

Botanical species	Common Name	Chinese name	% Of SFJD	Season of Harvesting	Place of Harvesting	Plant part	Processing	Extraction Solvent
*Reynoutria japonica* Houtt. [Polygonaceae]	Japanese Knotweed	Hu Zhang	16.67	Spring and Autumn	Anhui, Lu’an, China	rhizome	Washed, cut into thick slices, sun-dried then crushed	70% ethanol
*Forsythia suspensa* (Thunb.) Vahl [Oleaceae]	Weeping Forsythia	Lian Qiao	13.33	Autumn, when fruit is still green	Shanxi, Yuncheng, China	fruit	Steamed, sun-dried	Water
*Isatis tinctoria* subsp. *tinctoria* [Brassicaceae]	Indigo Woad	Ban Lan Gen	13.33	Autumn	Heilongjiang, Tsitsihar, China	root	Washed, sun-dried, cut and crushed	70% ethanol
*Bupleurum chinense* DC. [Apiaceae]	Chinese thoroughwax	Chai Hu	13.33	Spring and Autumn	Shaanxi, Weinan, China	root	Dried, washed, and cut into segments	Water
*Patrinia scabiosifolia* Link [Caprifoliaceae]	Yellow Flowered Valerian	Bai Jiang Cao	13.33	Before flowering in summer	Hubei, Enshi, China	herb	Half dried in the sun, bundled, then dried in shade, cleaned, cut into segments	Water
Verbena officinalis L. [Verbenaceae]	Vervain	Ma Bian Cao	13.33	When flowering in June - August	Hubei, Xiangyang, China	herb	Cleaned, sun-dried, cut into segments	Water
*Phragmites australis subsp. australis [*Poaceae*]*	Common reed	Lu Gen	10	Not specified	Hebei, Anguo, China	rhizome	Sun-dried, washed, cut, dried again	Water
*Glycyrrhiza uralensis* Fisch. ex DC. [Fabaceae]	Chinese liquorice	Gan Cao	6.7	Spring and Autumn	Gansu, Dingxi, China	root	Sun-dried, washed, cut, dried again	Water
Corn dextrin								
Silicon dioxide								

As the idea of giving Chinese Herbal Medicine for AECOPD is novel in the UK, it is not clear to what extent this would be feasible or acceptable to patients or clinicians. Therefore, we aimed to evaluate the acceptability of giving SFJD as adjunctive treatment in patients treated for AECOPD in primary care in the UK. We also aimed to assess the feasibility of conducting a full trial to assess the safety and effectiveness of adding SFJD to standard treatment for improving speed of recovery, reducing necessity for antibiotics and reducing risk of admission to hospital.

## Methods

The trial protocol has been published in full (Hu et al., 2022) so will simply be summarised here.

### Trial design

This was a double-blind, randomised placebo-controlled feasibility trial, incorporating a nested qualitative study. This study is reported following the CONSORT herbal extension checklist (Gagnier et al., 2006) and ConPhyMP guidelines (Heinrich et al., 2022), with the nested qualitative elements being reported using the COREQ checklist (Tong et al., 2007). Eligible patients were randomised in a 1:1 ratio to receive SFJD capsules or placebo capsules, in addition to the best current practice based on guidance for managing an AECOPD (National Institute for Clinical Excellence, 2019).

### Participants

Eight general practices in the Wessex region of the United Kingdom conducted a search and mail-out to all their registered patients aged 40 or over, with a documented diagnosis of COPD in their medical record. Patients were informed about the trial and asked to contact their general practice the next time they experienced an exacerbation so they could be assessed for the trial. Patients who were not willing to participate in the event of an exacerbation were invited to contact the trial team on a reply slip so that they could participate in a qualitative interview to understand the barriers to recruitment.

Participants were patients who experienced an Acute Exacerbation of COPD (AECOPD) and were willing to participate. AECOPD was defined as increased breathlessness, increased sputum purulence or increased sputum volume for at least 24 h and less than 21 days. The recruiting clinician had to be considering use of antibiotics, and the patient needed to provide informed consent and to be able to provide self-reported outcome data at 2 and 4 weeks. Patients were excluded if they were already taking antibiotics or corticosteroids, if they had another chronic lung disease (such as cancer or bronchiectasis) or if the responsible clinician diagnosed a severe illness and/or decided that an urgent hospital referral was needed.

### Intervention

Participants were randomised to receive either Shufeng Jiedu (SFJD, batch No. 3210501), or placebo identical in appearance and similar in taste. The dose was 4 × 520 mg capsules to be taken three times daily, preferably after meals, for 14 consecutive days. This is the standard adult dose for SFJD.

SFJD (manufactured by Anhui Jiren Pharmaceutical Co., Ltd.) was licensed as an over-the-counter drug in China in July 2021 (National Medical Products Administration, 2021). Its eight constituent herbs and their plant parts are listed in Table 1. Before the herbs enter the factory, professional and technical personnel check the macroscopic and microscopic characteristics of the eight medicinal materials and identify them according to the specifications in the Chinese Pharmacopoeia 2015 to ensure that the herbs are correct. In addition, laboratory analysis, such as High Performance Liquid Chromatography (HPLC), is performed on samples of the eight herbs at the company’s laboratory. A sample of each herb is retained and stored in the Central Laboratory Sample Retention Room at Anhui Jiren Pharmaceutical Co., Ltd. In addition, HPLC is used to ensure that the medicinal product contained sufficient levels of three reference compounds: emodin and polydatin (from *Reynoutria japonica*) and Phillyrin (from *Forsythia suspensa*).

The manufacturing process is as follows. *Reynoutria japonica* rhizome and *Isatis tinctoria* root coarse particles are put in 70% ethanol at a volume ratio of 5:1 ethanol: ground mixture. This is heated under reflux for 2 hours, then filtered. The sediment is mixed with 70% ethanol at a volume ratio of 3:1 ethanol: sediment, heated under reflux for 1 hour, and filtered. The filtrates are combined, ethanol is recovered, and filtrates are vacuum concentrated into a thick paste with a relative density of 1.35–1.40 (at 60 °C).

*Forsythia suspensa* fruit and *Bupleurum* root are placed in water, the volatile oil is extracted for 4 hours, and put aside. The mixture is then filtered, and both the filtrate and sediment are kept. The sediment is placed in water with *Verbena, Patrinia, Phragmitis* rhizome, and *Glycyrrhiza uralensis* root, and boiled for 2 hours and then again for 1 hour. This is filtered and combined with the filtrate from *F. suspensa* fruit and *Bupleurum* root, then vacuum concentrated into a thick paste with a relative density of 1.35–1.40 (at 60 °C). 50 g of dextrin and 50 g of micro-silica gel are mixed well with the two pastes. The mixture is vacuum dried, powderised and dextrin is added so that the total weight reaches 520 g. The volatile oil (diluted with appropriate amount of absolute ethanol) is sprayed into the mixture. The mixture is then sieved, mixed evenly, and packed into 1,000 capsules each of 520 mg.

The placebo capsule was made by the same manufacturer and was designed to mimic the odour and taste of the active medication. It contained corn dextrin (79.66%), caramel (4.62%), food additive lemon yellow (0.35%), compound colourant chocolate brown (0.05%), compound colourant gardenia yellow (0.19%), compound colourant Cocoa Brown (0.23%), naringin (9.62%), anhydrous citric acid (0.96%), menthol (0.96%), FA-10101 sauce flavour essence (2.88%), and MCK135C ginger powder base (0.48%).

### Outcomes

The pre-defined outcomes were as follows.

1) Recruitment process and retention
  • Eligibility: Proportion of patients on the COPD register who present with AECOPD
  • Eligibility: Proportion of AECOPD-presenting patients eligible and ineligible for the trial (plus reasons)
  • Recruitment/Randomisation: Proportion of eligible patients recruited/randomised
  • Recruitment: Rate of recruitment per month open in the UK primary care setting
  • Retention: Across the duration of the trial
2) Intervention management and procedures
  • Intervention adherence according to diary data and returned medication
  • Average no. of capsules taken per day per patient
  • Duration of treatment per patient
  • Safety and adverse drug reaction (ADR) reporting
  • Effectiveness of blinding: Proportion of patients correctly guessing treatment/placebo allocation and reasons why.
3) Completion of outcome measures
  • Proportion of diary completion (including a daily record of treatments taken and symptoms as measured by the EXAcerbations of Chronic pulmonary disease Tool - Patient-Reported Outcome (EXACT-PRO^®^) questionnaire (Choi et al., 2019). In addition, participants were asked to complete the COPD Assessment Test (CAT)™ symptom questionnaire (Gupta et al., 2014) at days 14 and 28).
  • Proportion of patients returning trial diaries
  • Proportion of patients who took antibiotics in each group
  • Proportion of patients given immediate and delayed antibiotic prescriptions

### Sample size

The sample size for this trial was 80 patients (40 per arm) over 12 months. As this was a feasibility trial, no formal comparative sample size calculation was carried out. Using a 95% confidence interval approach and an expected proportion of 50% (to give the worst-case scenario) it can be shown that this sample size would allow us to predict the recruitment rate to within 13% [IBM SPSS Statistics for Macintosh, Version 25.0].

### Consent

Written informed consent was obtained from all patients prior to confirmation of eligibility and randomisation into the trial.

### Randomisation

Patients were randomised to receive either SFJD or placebo capsules in a 1:1 allocation ratio. The randomisation sequence was generated using block randomisation with no stratification factors with Stata version 16.0 (StataCorp LLC) by a statistician at the Southampton Clinical Trials Unit (SCTU). Randomisation codes were securely sent to a labelling technician at Anhui Jiren Pharmaceutical Co., Ltd. China, who was not involved in the administration of the trial. The treatment packs were held at the primary care sites and were issued to participants following consent, confirmation of eligibility and randomisation.

### Blinding

Neither the GP, research nurse, or healthcare assistant allocating the treatment packs, nor the patient, knew to which arm they had been randomised. The treatment packs containing either SFJD or placebo capsules were provided in identical packaging to ensure blinding.

### Statistical methods

All analyses were conducted according to a pre-specified Statistical Analysis Plan. The ITT population included all randomised participants, regardless of treatment adherence or the actual treatment received. SAS version 9.4 was used for analyses. Data are described using descriptive statistics appropriate to the nature of the data. There were no formal statistical comparisons between groups.

Full medication adherence was assumed to be 4 capsules, 3 times a day over 14 days (4 × 3 × 14 = 168 capsules). Adherence with medication was calculated based on returned medication; if not available then from Participant Diary; and finally, if neither were available then from diary by recall.

Diary completion *(2 days confirmed symptom resolution)* was defined as Participant Diary completed up to 2 days after the complete resolution of symptoms or for 28 days, whichever was earliest. The symptom field had to be complete for all days within that period to count as completed overall. A diary was counted as complete irrespective of whether the data was completed in the participant diary or was completed later via diary by recall. All serious adverse events occurring up until 21 days post randomisation were reported on the trial specific SAE/SUSAR form and were reported to SCTU within 24 h of a site becoming aware of the event. All trial medication-related non-serious adverse events occurring up until 21 days post randomisation were recorded in participant diaries.

### Nested qualitative study

We invited all recruited trial participants, and all those who met eligibility criteria but refused to participate (as above), to take part in a remote semi-structured interview. In addition, we invited the recruiting GPs and nurses to be interviewed about their experiences. Interviews were conducted by telephone and were audio-recorded and transcribed verbatim.

The transcripts were analysed using both deductive framework analysis (Ritchie and Spencer, 1994) and inductive thematic analysis (Braun and Clarke, 2006) to understand barriers and facilitators to recruitment and participation in the trial. Once the transcripts had been read through for familiarisation, the data were coded using NVivo10 software. Initial themes were then generated and refined through continued reading and analysis and multiple coding and discussion with two co-investigators. Quotes were also extracted into frameworks in spreadsheets corresponding to the main themes. Themes were developed by recognising concepts directly communicated by participants, with subsequent consideration given to deeper connections and patterns when interrogating the findings.

### Ethical Approval

The trial and all subsequent amendments were reviewed, and favourable opinion granted by the London Surrey Research Ethics Committee (20/LO/0580) prior to opening recruitment or implementing any changes to the trial.

## Results

### Recruitment

The start of the trial was delayed by 2 years because of the COVID-19 pandemic and the need to amend the protocol to allow for remote consultations and recruitment. As a result of these delays the trial was only open to recruitment for 6 months instead of the planned 12 (from end of Jan to end of July 2022).

In total there were 2,285 registered COPD patients at seven practices (the eighth practice neither provided data on COPD patients, nor recruited any participants, so did not contribute to these figures). There was marked heterogeneity between the seven GP practices. The majority of patients (13) were recruited by just two practices, which had dedicated research staff, while another two practices recruited three patients each. Three practices recruited no patients at all (of which two screened 7 and 9 patients and one screened none).

**FIGURE 1:**
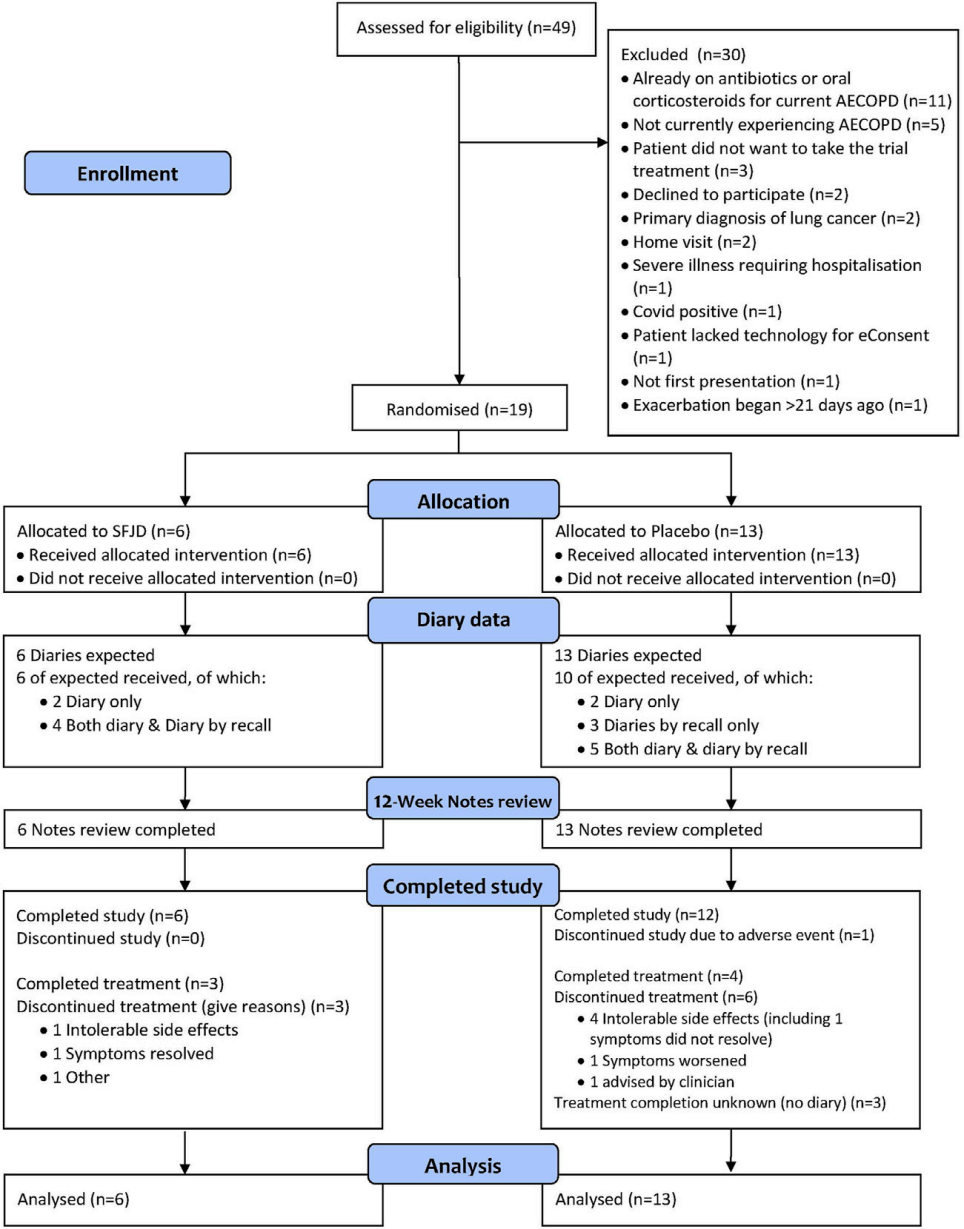
CONSORT flow diagram.

In total, 49 patients were screened. Only 44 were experiencing AECOPD and of these, five did not want to participate. Twenty were excluded for other reasons, most commonly because they had already started taking antibiotics or steroids from their “rescue packs” before seeing a GP. Nineteen patients were recruited into the trial (Figure 1). This translates into a recruitment rate of 1.3 participants per 1,000 registered COPD patients per month open.

### Patient characteristics

There were no large differences between the intervention and control group in demographic characteristics, past medical history, or presenting symptoms and signs of the current exacerbation (Table 2). Most patients had increased sputum purulence and/or increased breathlessness, but with normal oxygen saturation and normal temperature. None were on maintenance corticosteroids.

**TABLE 2:** Baseline characteristics of participants.

	SFJD (n = 6)	Placebo (n = 13)
Median age (IQR)	71 (68–79)	71 (68–77)
Women—n (%)	2 (33.3%)	6 (46.2%)
White ethnicity	6 (100%)	7/7 (100%)
Current smokers	3 (50.0%)	3/7 (42.9%)
Mean years of smoking (SD)	45.0 (20.25)	45.3 (11.64)
Any relevant past medical history—n (%)	4 (66.7%)	9 (69.2%)
Asthma—n (%)	1 (16.7%)	4 (30.8%)
Cardiovascular disease—n (%)	2 (33.3%)	1 (7.7%)
Hypertension—n (%)	4 (66.7%)	5 (38.5%)
Diabetes—n (%)	3 (50.0%)	3 (23.1%)
Median duration (days) of current exacerbation (IQR)	6.5 (3.0–14.0)	7.0 (3.0–14.0)
Increased sputum purulence—n (%)	5 (83.3%)	10 (76.9%)
Increased sputum volume—n (%)	4 (66.7%)	9 (69.2%)
Increased breathlessness—n (%)	5 (83.3%)	12 (92.3%)
Mean temperature (SD)	36.6 °C (0.47)	36.6 °C (0.36)
Median SpO2 (IQR)	96.0% (95.0—97.0)	97.0% (95.0—97.0)
Mean CAT score (SD)	23.5 (7.82)	29.3 (7.30)
Mean EXACT-PRO score (SD)	50.3 (10.11)	53.6 (9.74)

### Randomisation

Six patients were randomised to SFJD and 13 patients to the control, representing an allocation ratio of 1:2 rather than the 1:1 expected. Contributing factors include the low recruitment rate at individual sites and the removal of treatment packs that were damaged in transit from the Anhui Jiren factory in China to the UK. Despite the removal of additional packs to balance the randomisation blocks and a review of allocation at 10 patients (which showed that allocation was 1:1 at that point in time), by chance the following 9 patients were predominantly provided with treatment packs containing placebo.

### Outcomes

#### Retention and completion of outcome measures

All patients in the SFJD group and ten (77%) in the placebo group returned their diaries (either on paper, or by recall, or both) (Figure 1 and Table 3). Participants completed their diaries for a median of 11 days (IQR 8–21 days) in both groups. The 12-week notes review was completed for 100% of participants in both groups (n = 19).

**TABLE 3:** Completion of outcome measures.

Outcome measure	SFJD (n = 6)	Placebo (n = 13)
Trial diary returned—n (%)	6 (100%)	7 (53.8%)
Proportion of trial diary completed—n (%)		
• Complete Diaries (7 days confirmed symptom resolution)	5 (83.3%)	6 (46.2%)
• Complete Diaries (2 days confirmed symptom resolution)	1 (16.7%)	1 (7.7%)
• Incomplete Diaries with useable antibiotic use data	0 (0%)	3 (23.1%)
• No Diary returned	0 (0%)	3 (23.1%)
Mean number of days on which complete diary data was entered (SD)	13.7 (9.07)	13.7 (8.85)
No of patients providing complete useable endpoint data at 28 days—n (%)^1,4^	0 (0%)	0 (0%)
• Complete useable trial medication data	3 (50.0%)	3 (23.1%)
• Complete useable antibiotic data	5 (83.3%)	9 (69.2%)
• Complete useable steroid data	2 (33.3%)	2 (15.4%)
• Complete useable symptom resolution data	6 (100%)	8 (61.5%)
• Complete useable EXACT-PRO data	3 (50.0%)	2 (15.4%)
• Complete useable CAT data	4 (66.7%)	4 (30.8%)

#### Adherence and side-effects

There was a very wide range of treatment adherence in both groups (from 21.4% to 100% in the SFJD group and 19.0%–100% in the placebo group) (see Table 4). However, overall adherence appeared to be better in the placebo group (median 77.4%) compared to the SFJD group (median 54.8%). The median number of capsules taken per day was 12 in both groups, but median duration of treatment in the SFJD group was shorter than in the placebo group (9.8 vs. 13.3 days). Of the 9/15 (60%) who discontinued early, the median duration was 6.3 days (IQR 3.3–8.3).

**TABLE 4:** Adherence and side-effects.

	SFJD (n = 6)	Placebo (n = 13)
Overall adherence to medication - median % of capsules taken (IQR)	54.8% (38.1—100.0)	77.4% (36.9—100.0)
Mean number of capsules taken per day (SD)	11.3 (1.44)	11.2 (2.36)
Median days of treatment (IQR)	9.8 (5.3–14.0)	13.3 (7.0–14.0)
Reported side-effects to trial medication in participant diary in Week 1—n (%)	2 (40.0%)	3 (50.0%)
Correct treatment allocation guessed—n (%)	2 (50.0%)	0 (0%)

Four patients in each group completed the treatment or stopped it because their symptoms had resolved. Four (40%) in the placebo group and one (17%) in the SFJD group stopped because of perceived intolerable side-effects. In the first week, two participants in the SFJD group and three in the placebo group reported side-effects, but more stopped the treatment because of “intolerable side-effects” in the placebo group. One patient on SFJD had an episode of stomach pain, and another experienced rectal bleeding but after stopping the treatment, felt this was unrelated to the treatment. In the placebo group, one patient reported nausea, one experienced melaena and one developed high blood pressure so was advised to stop the treatment by their GP.

One patient on placebo (and none on SFJD) discontinued the study because of an adverse event (hip fracture requiring hospital admission). In total, adverse events were reported by one participant in the SFJD group (mild upper abdominal pain) and by 3 participants on placebo–two moderate (one lower respiratory tract infection–thought to be unrelated to the treatment; one tachycardia–judged to be related to the treatment, so treatment stopped) and one severe (hip fracture - unrelated).

#### Blinding

Blinding was effective, as no patients in the placebo group correctly guessed their treatment allocation, and only 2 in the SFJD group guessed that they were receiving the active treatment.

#### Use of antibiotics and steroids

Immediate antibiotics were prescribed for three patients in the SFJD group and six in the placebo group, with a further two in each group receiving a delayed prescription (see Table 5). This was most commonly doxycycline, followed by amoxicillin. Four patients in each group took the antibiotics starting on the first day. A further two patients in the placebo group (and none in the SFJD group) took antibiotics at day 2 and day 4.

**TABLE 5:** Use of concomitant treatments.

	SFJD (n = 6)	Placebo (n = 13)
Patients prescribed antibiotics at initial consultation—n (%)	5 (83.3%)	8 (61.5%)
• Immediate	3 (50.0%)	6 (46.2%)
• Delayed	2 (33.3%)	2 (15.4%)
Patients who took antibiotics within 28 days of entering the study—n (%)	4 (66.7%)	6/10 (60.0%)
Oral corticosteroids prescribed—n (%)	4 (66.7%)	8 (61.5%)
Patients who took oral corticosteroids within 28 days of entering the study—n (%)	4/4 (100%)	4/4 (100%)

#### Symptom duration and severity

**FIGURE 2:**
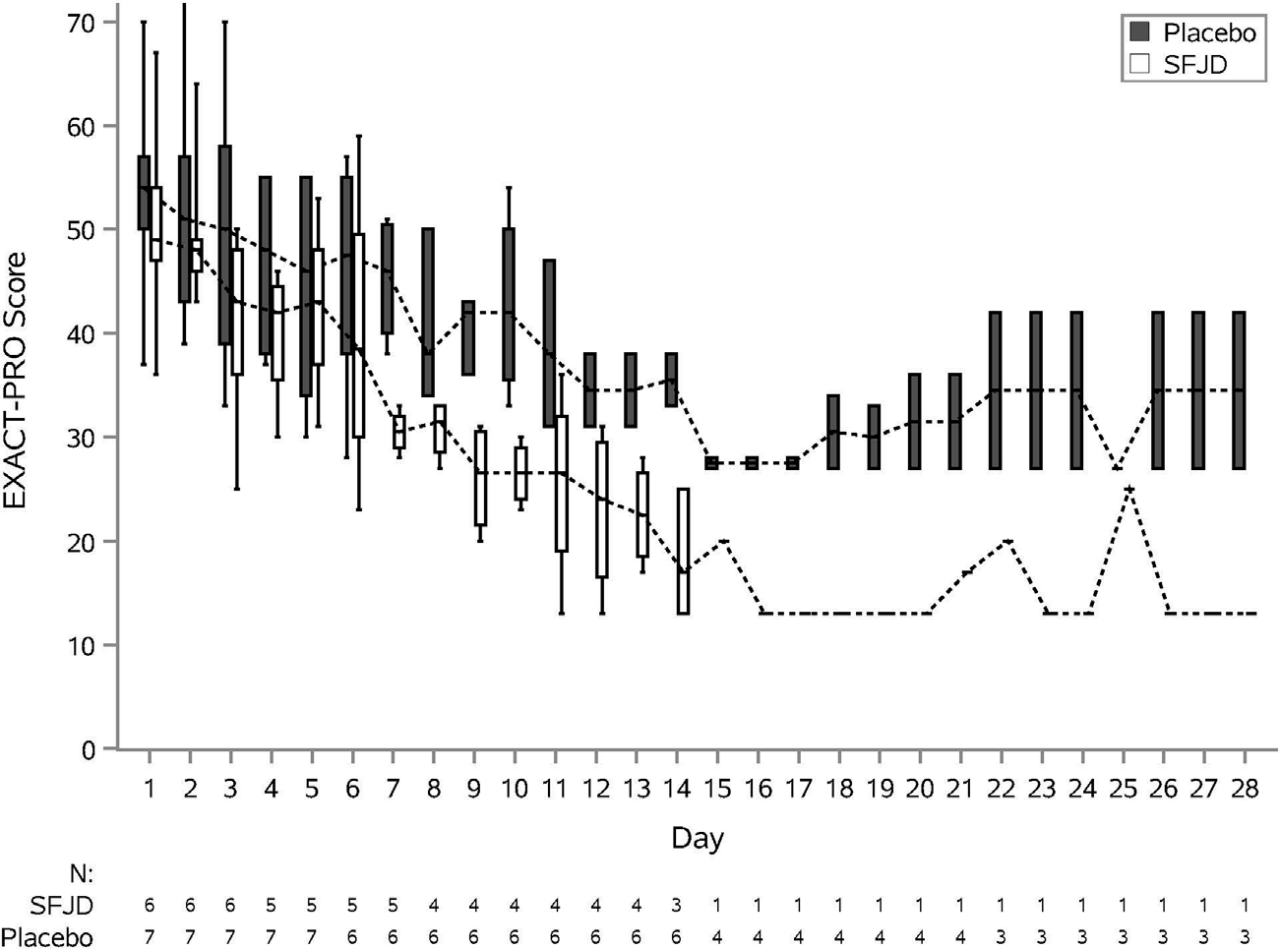
EXACT-PRO Scores: Plots from baseline (day 1) to day 28, by treatment arm.

**FIGURE 3:**
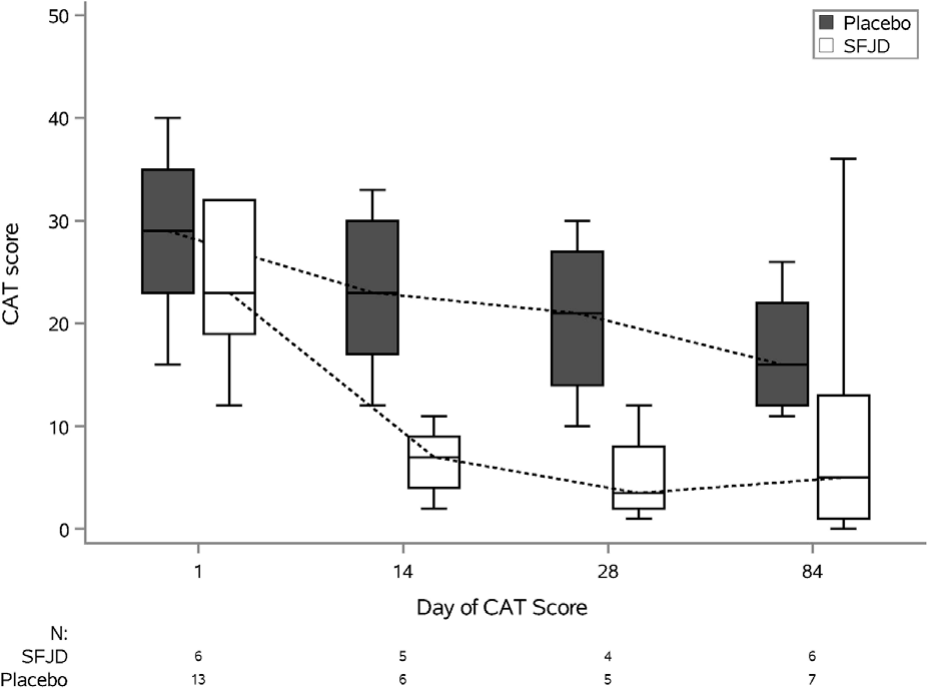
COPD Assessment Test (CAT) Scores: Plots from baseline (day 1) to day 84, by treatment arm.

We piloted several measures of symptom duration and severity (Table 6; Figure 2, Figure 3). Median first day of symptom resolution was 7.5 in the SFJD group and 6 in the placebo group. We did not design (numbers were insufficient) to test for statistically significant differences between the groups. However, the distribution of patient reported outcome measure scores over time suggest possible small benefits of SFJD (Figure 2, Figure 3).

**TABLE 6:** Symptom -related outcomes.

	SFJD (n = 6)	Placebo (n = 13)
Median first day of symptom resolution (IQR)	7.5 (7.0–9.0)	6.0 (3.5–8.5)
Median duration of episode in days (IQR)	13.5 (11.0–20.0)	11.0 (10.0–15.0)
No of participants with at least one AECOPD-related primary care consultation within notes review period—n (%)	2 (33.3%)	4 (30.8%)
No of participants with at least one hospital outpatient visit related to AECOPD within review period—n (%)	1 (16.7%)	2 (15.4%)
No of participants with at least one hospital admission related to AECOPD	0 (0%)	2 (15.4%)

#### Nested qualitative study

Eleven (n = 11/19) Excalibur trial participants also consented to be contacted for a qualitative interview. Of these, five were interviewed while one was non-contactable and five rejected the invitation. They were unaware of their treatment allocation at the time of the interviews. Of the five recruited participants, after unblinding it transpired that only one had been taking SFJD while four had taken placebo.

Regarding the five patients who were approached but declined to participate in the trial, all refused to consent to an interview. However, we interviewed non-participants identified through the mail-out to the registered COPD patients. In response to the mail-out, 77 consented to be contacted about an interview. Of these, only 23 said they would not have been willing to participate in the trial, of whom 12 were uncontactable and 5 declined the invitation. In total six non-participants consented to interview. We also interviewed nine clinicians involved in the delivery of the Excalibur trial (Table 7).

**TABLE 7:** Characteristics of participants in the nested qualitative study.

Group	Non-recruited patients	Recruited participants	Staff
Gender			
Male	4 (67%)	3 (60%)	1 (11%)
Female	2 (33%)	2 (40%)	8 (89%)
Median age (IQR))	77 (71–78)	69 (66–79)	49 (28–53)
Ethnicity			
White	7 (100%)	5 (100%)	7 (78%)
Asian	0	0	1 (11%)
Mixed	0	0	1 (11%)

#### Reasons for low recruitment and suggestions for improving recruitment

Firstly, the timing of the trial was unfortunate because it missed much of the winter period when exacerbations are most common (Wilkinson et al., 2017), and also many COPD patients were still being exceptionally careful to avoid social contact in the wake of the COVID-19 pandemic, so the incidence of exacerbations was much lower than would normally be expected (Table 8). Two non-recruited patients and several clinicians mentioned that several people were interested to take part but did not experience exacerbations during the recruitment period (and some therefore felt that the invitation letter was not appropriate for them).

**TABLE 8:** Reasons for low recruitment and suggested solutions to improve recruitment.

Reason for low recruitment	Example quote	Suggestion	Example quote
Patients not experiencing exacerbations	“*It was so long ago* … *I don’t have a recent example of exacerbation - I can’t even say the word! - but a recent example for it to be the subject of study so that’s the reason I declined to take part*.” (85 year old male COPD patient, non-participant)	Longer recruitment period, including over the winter	*“It needs to run over the winter months. There’s no way that you can recruit to something where you’re looking for viral illnesses or exacerbations over the summer months. You’re just not going to find the levels that you need*.” (28 year old female research nurse, recruited 0 patients)
	“*Actually, a lot of people would phone in and say, ‘Oh, I’m really interested in taking part,’ and they were not having an exacerbation at the time so they couldn’t*.” (28 year old research nurse, recruited 3 participants)		
	“*I mean the problem I had with that trial was just getting patients, because everybody was locked down and staying at home, they weren’t really going out, so they didn’t really get exacerbations*.” (52 year old female GP, recruited 7 patients)		
Patients use “Rescue” packs at home before seeing GP	“*This is a very different population and unfortunately the way that this population works is that they have these rescue packs at home, and therefore they have the ability just to take their medicines as and when they do, and it’s quite a learnt behaviour over a long time*.” (28 year old research nurse, recruited 0 participants)	Include trial medication as a “standby pack”	“*Well, I guess if you’ve got your COPDs that do exacerbate fairly regularly - particularly over the winter - you could prep them all in the summer or autumn to say, ‘You could have this at home. You can have a rescue pack at home.’* … *They could have the drugs at home and call and get randomised, but then … Yes, so they could still see the GP for a medical assessment*.“.” (46 year old practice nurse, recruited no participants)
Challenge of opportunistic recruitment of acutely ill patients	“*The problem with opportunistic trials is having the person who’s trained there at the time that can put that person in the trial. I did put these people in the trial myself, but I really had to just drop everything and put them in the trial, which just makes other patients have to wait, or you have to make the participant wait until you get to the end of the morning. It’s just not ideal, it really isn’t*.” (52 year old GP, recruited 7 patients)	Reduce workload for GPs by getting CRN nurses or CTU team to take consent and other procedures after the GP has identified the patient	“*The GPs will, but they just have to press a button and it brings up everything they need to do and they just do some tick boxes. Then the study site does the rest, i.e., consenting, sending the drug, etc. It’s got to be really quick, really easy in GP land.* … *You need to offer staffing, you need to make it as easy as possible. You need to make it time-wise as short as humanly possible. You need to offer - you need to make it an appealing package in support, i.e., can the consents be done by the central team, can the follow-ups be done by the central team? Can the drugs be posted out to the patient? Is there a helpline the patient can ring to get advice about the study and discuss it?*” (46 year old female practice nurse, recruited 0 patients)
	“*So recognising, finding the patient is one thing but then having time to do something, to carry out the research there and then to recruit them, I would honestly say it’s getting almost impossible to do that now*.” (28 year old research nurse, recruited 0 patients)		
Too time-consuming for patients and clinicians	“*We had a few patients that declined it because it would have involved coming into the surgery for an hour, an hour and a half to go through everything and they just didn’t want to do that*.” (28 year old research nurse, recruited 3 patients)	Simplify trial procedures and go paperless	“*I’ve done another study that only required ECRFs, then now I say we can just stick to the ECRF, we do not need a paper one and an electronic one. At the beginning we were a bit sceptical about how everything’s going to work, whether it’s all going to flow properly, but now that we’re doing other studies and we’re relying completely on the ECRF, I would say I think we just do ECRF. We do keep that option open in terms of if anyone can’t do the ECRF, is someone having technical difficulties, but just to rely on that would save quite a lot of time I think*.” (28 year old female trial coordinator, recruited 7 participants)
	“*There was an awful amount of paperwork and it was a huge burden on the amount of time that it took. … . It took me about 4 hours to do the first patient which was absolutely ridiculous*.” (53 year old research nurse, recruited 7 patients)		
Insufficient information on the trial medication and procedures	“*It didn’t give me any details of what had gone before, what the experience in Southeast Asia had been, why it was thought it might have some benefit in the UK.* … *Yes, and what led the university to deciding this was worth following up? It’s almost as if the university decided we’re the university, and if we approach somebody because we’re the university, they’ll think we’re on it, and we don’t need to tell them any more*.” (78 year old male COPD patient, retired engineer, non-participant)	More detailed information on the trial medication	“*I don’t just believe things like that without checking them out. I do the same thing with the drugs I get too.* … *Generally speaking, I would take the practitioner’s view, obviously, but if I’ve got any concerns I would be in there looking at exactly what it is. I’m one of these guys that reads all of the leaflets that comes with the tablets once, then keep it in my file* … … *some of this has come through recommendation. Some of it’s come through reading, and also the comparisons, one with another, which also helps. I’m not sure with herbal exactly - it has to be something which you think you’re going to get benefit from*.” (58 year old male COPD patient, non-participant)
	*“Taking part in a study which has a double-blind in it means that the odds of success are much reduced because if I’m one of the placebo group, then I would not be getting any treatment, and consequently, for me, at my stage in life, that’s too much of a risk. That was the real cards on the table reason why I did not want to get involved.”* (78 year old retired COPD patient, non-participant)		“*Maybe having a little bit more training on how does it work, maybe about the studies as well, in China, and why is it doing that. If I knew that, I could be more confident in selling it. There is quite a lot of things that they were not known to me, so I was not able to pass it on completely*.” (28 year old research nurse, recruited 7 participants)

Secondly, the biggest challenge for GP practices was finding capacity in an already overstretched workforce to opportunistically recruit patients with an acute illness. Several recruiting clinicians also referred to the administrative burden and time taken to complete all the paperwork for the trial. Many GP practices simply did not have enough staff to devote to research; and unless there were dedicated research staff, studies such as these would be impossible to run. Several suggested following the example of other trials such as ATHENA (https://www.isrctn.com/ISRCTNISRCTN14490832) in which the GPs simply identify suitable patients, and the central trial team does the rest of the work.

Thirdly, many COPD patients have “rescue packs” of antibiotics and steroids, which they start before even contacting their GP. The largest reason for excluding potential participants was that they had already started antibiotics and/or steroids. One of the research nurses suggested that the trial medication could be included in “rescue packs” for patients who experience frequent exacerbations.

Fourth, several patients expressed the wish to see more detailed information about the trial medication and others clearly had not understood that it would be given alongside their normal treatments (because they were unwilling to risk being given a placebo). Herbal medicine was not a barrier to recruitment for patients or clinicians, although two patients said they were sceptical about it and one did not want to take any Chinese medicine (because of worries it could contain animal substances).

#### Views on the trial medication

The recruited patients were happy to have been approached through their GP surgery when they presented with an exacerbation. In addition to discussing the trial with the GP surgery and trial coordinating centre staff, all trial participants appreciated being provided with written information and felt that they understood what was being offered. Most also expressed a motivation to help find better treatments, both for themselves and for others.

*“I just wanted to try something different because, as I say, I'd been ill for so long and I thought, it would be worth giving this a try and if it worked, wouldn’t it be great, not just for me but for future people, other people that are poorly with COPD?”* (79 year-old female participant)

Although most of the participants had never taken herbal medicines before, they were open to try anything which could help. Almost all seemed daunted by the quantity of capsules, especially as most were also taking many other medications for co-morbidities. Nevertheless, all interviewed participants reported that they managed to fit them into their schedule and remembered to take them. Of note, all were retired and one commented he could not have managed it if he had been working.

“*If you was working, there's no way you'd manage them four times a day tablets, if you were going off to work at seven in the morning. That would be another - I was at home, it did not bother me at all, I had nothing to do. If you were working 12-hour shifts, or even eight-hour shifts, that would be a tie, all those tablets.”* (66-year-old retired male participant, placebo group).

However, most stopped taking the medication early because of perceived side-effects (although most of these were actually taking placebo). Most recruited participants understood the reason for being put on a placebo and accepted this: although some people refused to take part because they didn't want a placebo (Table 8).

“*When you think it could have been a placebo I've taken. I don’t give a monkey, because if it was a placebo, I certainly felt better for it.”* (66-year-old retired male participant, placebo group)

#### Diary completion and follow-up

Most interviewed participants found the diary simple to complete and had no suggestions for improving it. One mentioned that he would not have been able to complete forms online, and preferred paper. Participants appreciated the phone calls from the study team.

#### Outcome measures

For almost all patients, the most important outcome was whether they were able to do their normal activities, such as walking, singing, gardening or even sleeping. Most of these became more difficult or impossible during an exacerbation, mainly because of breathlessness. A few participants also mentioned general tiredness, sputum production and cough.

## Discussion

### Summary of main findings

Although the trial recruited fewer patients than planned, the reasons for this are clear and remediable. Most importantly, the idea of trialling a Chinese herbal medicine instead of antibiotics for AECOPD was widely acceptable to the majority of patients and clinicians. The diary and outcome measures were well completed and returned by the majority of participants.

Regarding the choice of primary outcome measure, patients interviewed were most interested in being able to return to their daily activities. This was measured by the EXACT-PRO questionnaire. This may be the most meaningful and sensitive primary outcome measure rather than total duration of any symptoms. As many patients now hold “rescue packs” it may be difficult to demonstrate any reduction in antibiotic use, unless the trial medication is included in the “rescue pack” and patients are advised to try it first for a few days, and only to take the antibiotics if they do not improve.

A future trial should have longer, over-winter recruitment periods, should use the central team to alleviate the burden of recruitment on busy GPs, and should consider including the trial medication as part of “rescue packs” which patients hold at home. More detailed information about the herbal medication should also be provided.

### Comparison with existing research

Although there have been many trials of herbal medicine for AECOPD in China, this is the first in the UK, where the population is much less accustomed to using herbal remedies. Nevertheless, our findings confirm results of previous research in acute bronchitis and other acute respiratory infections, which showed that most patients and clinicians are willing to try herbal alternatives to antibiotics (Soilemezi et al., 2020; Willcox et al., 2020; Willcox et al., 2021).

### Strengths and limitations

The trial was severely delayed and the recruitment time was curtailed due to the COVID-19 pandemic and funding constraints. This limited the timeframe for recruitment and also limited the number of patients meeting inclusion criteria. Randomisation was unequal by chance, because of the small sample size and some trial medication packs being damaged in transit, but this would be unlikely in a larger randomised trial. Also by chance, most of the qualitative interviews were with patients who had been randomised to placebo. However, this should not have affected their views about the trial or the medication, because none of them accurately guessed their randomisation group. A limitation was that all participants in the qualitative interviews (and in the trial) were of white ethnicity, so it is unclear whether the same findings would apply to other ethnic groups.

As social mixing returns to normal patterns and the incidence of AECOPD returns to normal levels, more patients will meet the inclusion criteria. A strength of the trial was that Good Clinical Practice was followed very rigorously. Although not being labelled as a Clinical Trial of an Investigational Medicinal Product (CTIMP) by the Medicines and Healthcare products Regulatory Agency (MHRA), this feasibility study was undertaken following CTIMP procedures, as requested by the trial sponsor, with the preparation of essential documents - IMP dossier, placebo dossier and a full safety dossier, and undertaking quality control testing of the trial medication after transit. This made it excessively time-consuming for many GP practices who did not have dedicated research staff, so alternative approaches need to be found to alleviate the administrative burden on busy GPs while facilitating opportunistic recruitment.

### Future research

The findings of this trial justify a fully-powered randomised controlled trial of SFJD for adjunct treatment of AECOPD in British general practice. There are clear recommendations for overcoming the challenges in recruitment which we faced. A future trial should include several full winter seasons. Since the COVID-19 pandemic, remote consultation and recruitment have become commonplace, including provision of pulse oximeters at home. Remote recruitment to trials has also become mainstream in trials such as PANORAMIC (Butler et al., 2023)—this methodology would lessen the workload for GP practices and would increase recruitment while still meeting GCP requirements. Perhaps the most straightfoward would be to include the trial medication as part of “rescue packs” for patients who experience frequent exacerbations, while keeping the trial open for a longer period and involving more regions of the UK. The main challenge is that this would require more patients to be recruited than would actually end up experiencing AECOPD during the trial period. The other main challenge is that although SFJD is manufactured according to Chinese GMP, it does not yet have EU/UK GMP and so does not yet meet the MHRA requirements for a CTIMP (Clinical Trial Investigational Medicinal Product).

If the future full-scale trial confirms that SFJD reduces the duration of exacerbations, reduces need for antibiotics, or reduces the risk of hospital admission, this will have several benefits for patients. Firstly, the reduction in antibiotic courses and reduction in hospital admissions and duration of hospitalisation will have a direct effect on reducing associated healthcare costs. Secondly, these effects would be expected to lead to a reduction in the development of antimicrobial resistance. Thirdly, reduction in duration of exacerbations and hospital admissions would also reduce the indirect costs of COPD due to sick leave. If found to be effective as hoped, SFJD could also then be used for the treatment of AECOPD in other countries, where COPD is becoming increasingly prevalent.

## Conclusion

Most COPD patients and their clinicians were happy to trial a Chinese herbal medicine as an alternative to antibiotics for the treatment of COPD. Recruitment to this trial was less than hoped because of challenges primarily caused by the COVID-19 pandemic. These challenges are remediable so a future trial should be feasible.

## Data Availability

The datasets presented in this article are not readily available because the participants did not consent for their data to be used in further research. Access to this data is strictly controlled by the Southampton Clinical Trials Unit and no third parties will be granted access to the Medidata servers holding research data. For any queries please contact ctu@soton.ac.uk.
